# The Use of Alcohol in Sudden Illness

**Published:** 1897-01

**Authors:** 


### Selections and Abstracts.

### THE USE OF ALCOHOL IN SUDDEN ILLNESS.

We have recently been asked (writes Ezra M. Hunt, M. D.) what are the best substitutes for alcoholic liquors in cases of sudden sickness. First of all, it is to be noticed that there is a great lack of discretion in dealing with persons who suddenly fall or become speechless. It is nearly always called a faint or a swoon, and somebody must run for the brandy bottle as if it were the only resort in such cases.

The fact is that we first have to determine whether the person is suffering from apoplexy, from sunstroke, or prostration by heat, from a hysterical attack, or even in some cases from feigned sickness. In all such instances it is now well understood that a stimulant is not needed. It has long since been recognized that all congestions about the brain need coldness to the head, and not increase of heart action. Sunstroke was once treated with stimulants, but now at the first these are carefully avoided. It is chiefly in cases of faintness from heart-failure, or shock, or from sudden pain, that stimulants are needed. Because bystanders are so apt to err in their diagnosis, those taken suddenly ill are apt to fare best if placed in a recumbent posture, with the head slightly elevated, if all tightness of garments about the neck or waist is relieved, and if a little cold water is given in case of ability to swallow. A mustard plaster on the back of the neck, or over the stomach, and hot water, or hot bottles to the feet, are never out of place; while vinegar, or smelling salts, or dilute ammonia, to the nostrils is reviving. It is thus seen that the call for any stimulant in the hands of the laity is rare.

We now come to speak of those cases in which there is need of a stimulant. If a physician is at hand, and the case is one of extreme heart failure, he will probably use a hypodermic syringe with a mixture of brandy and digitalin, since that is more speedy in its action. If brandy or other liquor is indicated, he will probably only use it briefly, and substitute it by aliments or slight sedatives. Dr. Richardson, of London, has insisted that in such cases we should not use the ethylic or common alcohol, but some of the other alcoholic series as equally available and not objectionable on moral grounds. We have found Hoffman's Anodyne a good substitute and do not know that it, with coffee or some of the peptonoids, will not accomplish all that liquors will.

Long ago our good professor taught us that in extreme faintness, especially from loss of blood, for an adult, five drops of laudanum in a half tablespoonful of brandy, given in water every fifteen minutes, until two or three doses are taken, is much better than any pure spirit. There are but very few cases of faintness in which, if the person is able to speak, a teaspoonful of paregoric in water is not of service, especially in cases of pain. It has in it benzoic acid, a slight stimulant; camphor, a stimulant; and then a nervine; and just enough of opiate to act as a stimulant first and sedative afterwards. If there is any one article to be recommended as of most universal application in the hands of bystanders, where there is ability in the patient to speak and swallow, we think this is the one. It should be given in a tablespoonful of water, a teaspoonful of the mixture being offered at first, so as to be sure as to capacity for swallowing. Carbonate of ammonia in powder, five grains to a half tablespoonful of water, is a good diffusible stimulant.

The promiscuous plan of giving alcoholic stimulants is really, in a large degree, the force of habit, and has no foundation in modern science or in good practice. It has survived from those days in which people took rum and applejack to keep out the cold, to keep out the heat, to help the appetite, to take the place of food, to wish health to a friend, to keep up the spirits; in fact to do anything that anyone who liked its pleasant taste wished to imagine.

We do not mean by this to say that alcohol is not available as a medicine. We believe, as such, it has a very important, although a narrow, sphere in the hands of the skilful and conscientious practitioner. It may also be of service in other hands, to meet sudden shock, where nothing else is at hand. But what we insist upon is, that it is very rarely useful for sudden illness or pain, in the hands of the public, to a degree that should give it preference over other remedies. Take, for instance, this matter of stimulation: it is by as distinguished an authority as Professor Lauder Brunton, of London, who tried the following experiment: He had a person who sipped a cup of hot water, and afterwards, at a proper interval, tried also the effect of a tablespoonful of brandy. In each case the sphygmograph was applied to the pulse at the wrist. It was found that the hot water increased the wave and rhythm of the pulse, and so affected the heartbeat as much as the brandy. The reviving effect of a teacupful of hot water with a teaspoonful of sugar, and six or eight drops of peppermint to give flavor, is very perceptible. This is really much of the value of catnip or other herb teas. A good prescription either in case of faintness or of pain is ten drops of spirits of camphor, ten drops of paregoric, ten drops of sweet spirits of nitre, and a half teaspoonful of baking soda in a tablespoonful of warm water. Ginger tea, made by putting a half teaspoonful of ordinary powdered ginger in a half teacupful of warm water, is a good stimulant. We doubt whether anything is gained in the interest of persons taken suddenly ill by suggesting a longer list of remedies. Position, cold to the head, stimulants to the nostrils, a mustard plaster, warmth to the feet, looseness of garments, and one of the stimulants we have named, followed by a drink of warm peppermint water, will do less harm and relieve more cases than dependence on the liquor flask. While not expelling alcohol from the physician's list of remedies, as a medicine as well as a drink, it has great abuse in the hands of promiscuous prescribers. While fully recognizing all that is necessary as to it, it surely behooves us to guard against excuses for its employment. All the more, because in some of these cases of sudden illness the person and his habits are not known to us.—*The Indian Lancet.*

---

The physicians of several of the cities in the United States having been aroused by reports of suffering in the Cuban papers, have started subscription lists to buy medicines, etc. The movement was inaugurated by the physicians of St. Paul. A committee, consisting of Drs. Kelly, Wheaton and Boeckman, have raised several hundred dollars for this purpose. It is expected, if necessary, that the supplies and medicines contributed can be sent under protection of the Red Cross Society, as no articles contraband of war will be sent.

